# PROMETHEUS: A Copper-Based Polymetallic Catalyst for Automotive Applications. Part II: Catalytic Efficiency an Endurance as Compared with Original Catalysts

**DOI:** 10.3390/ma14092226

**Published:** 2021-04-26

**Authors:** Iakovos Yakoumis, Εkaterini Polyzou, Anastasia Maria Moschovi

**Affiliations:** MONOLITHOS Catalysts & Recycling Ltd., 11476 Athens, Greece; kpolyzou@monolithos.gr (E.P.); moschovi@monolithos.gr (A.M.M.)

**Keywords:** PROMETHEUS catalyst, copper, catalytic converter, platinum group metals, automotive

## Abstract

PROMETHEUS catalyst, a copper-based polymetallic nano-catalyst has been proven to be suitable for automotive emission control applications. This novel catalyst consists of copper, palladium and rhodium nanoparticles as active phases, impregnated on an inorganic oxide substrate, CeO_2_/ZrO_2_ (75%, 25%). The aim of PROMETHEUS catalyst’s development is the substitution of a significant amount (85%) of Platinum Group Metals (PGMs) with copper nanoparticles while, at the same time, presenting high catalytic efficiency with respect to the commercial catalysts. In this work, an extensive investigation of the catalytic activity of full scale PROMETHEUS fresh and aged catalyst deposited on ceramic cordierites is presented and discussed. The catalytic activity was tested on an Synthetic Gas Bench (SGB) towards the oxidation of CO and CH_4_ and the reduction of NO. The loading of the washcoat was 2 wt% (metal content) on Cu, Pd, Rh with the corresponding metal ratio at 21:7:1. The concentration of the full-scale monolithic catalysts to be 0.032% total PGM loading for meeting Euro III standard and 0.089% for meeting Euro IV to Euro VIb standards. The catalytic activity of all catalysts was tested both in rich-burn (λ = 0.99) and lean-burn conditions (λ = 1.03).

## 1. Introduction

The increase of vehicle fleet during the last few decades has been linked with serious environmental impacts such as climate change and the greenhouse effect. Additionally, the increased population of the large urban centers is linked to the increased use of vehicles, one of main causes of air pollution in large cities. One of the precautionary measures to improve and reduce pollution from the use of fossil fuels is the strictest and lowest emission limits for cars. For many years, catalytic converters have been an effective way to reduce harmful gases emitted by internal combustion engines. Stricter emission limits for vehicles set in recent years have led to the development of new catalysts, which are characterized by higher concentrations of PGMs, compared to former generation of catalysts. The demand for higher quantities of PGMs to produce state-of-the-art catalysts also reveals an important economic aspect of the issue. The price of PGMs has increased significantly in the past 5 years (+165.68% for platinum, +467.26% for palladium and +1490.30% for rhodium) [1], while their supply is dependent on few producers and at the same time their substitution remains difficult. It is, therefore, easy to understand that the development of new catalytic systems that combine high efficiency with lower use of PGMs is a challenge for the research community, both for environmental and economic reasons [2].

An ideal new catalyst would be one that could combine high reactivity with one metal or a combination of metals characterized by high physical abundance and low cost. Copper, which has been increasingly used in catalytic applications over the last two decades, could be regarded as a proper candidate. The high natural abundance of copper, compared with precious metals, the interesting applications in organic synthesis (e.g., Gilman reagents or as co-catalyst in Sonogashira reaction) and its catalytic properties, is an attractive background [3,4,5]. The increased research interest and the development of new catalytic systems-based on copper is reflected in the multiple publications and the variety of catalytic applications that include homogeneous and heterogeneous catalytic systems. New homogeneous catalytic systems-based on copper catalysts presenting remarkable results have applied successfully in organic synthesis reactions. In many cases, the aim of the researchers is to replace palladium or precious metals catalysts with copper catalysts, e.g., copper catalyzed Sonoghasira coupling reaction [4,5]. These efforts are extended to even more sophisticated reactions such C–H activation reactions [6,7]. On the other hand, heterogeneous copper-based catalytic systems demonstrate extremely interesting catalytic properties, especially in oxidation reactions. Various catalytic systems of supported copper catalysts have been applied successfully in the oxidation of CO [8,9,10,11,12,13,14,15,16,17,18,19].

Pacella et al. presented nanocomposites in which CuO nanoparticles were highly dispersed on active LaCoO_3_ by an Ammonium-Driving-Deposition participation (ADP) procedure. The aim of the above research was the development of Platinum Group Metal (PGM) free catalysts for applications as Three-Way Catalytic Converters (TWC). Catalysts’ performance was tested in conditions approaching the effective automotive exhaust. A high efficiency with 85–90% CO conversion was observed at 250 °C and 100% NO conversion at 400 °C, under fuel-rich conditions [20]. Ce–Cu and Ce–Zr–Cu oxide systems with a flower-like morphology (FCCu and FCZCu, respectively) have been synthesized by slow co-precipitation method and calcined in air flow at 650 °C. The samples produced were characterized with various methods and were used as catalysts for the preferential oxidation of CO in excess of H_2_. The catalytic experiments took place in 80–190 °C temperature range. Both catalysts showed similar catalytic behavior below 140 °C, maintaining high CO conversion levels above 160 °C with FCZCu catalyst activity remaining constant at about 100% up to 190 °C. The selectivity to CO_2_ over the FCCu catalyst was sharply decreased above 90 °C with the exception of FCZCu that remained selective up to 115 °C (98%) [21].

CuO/CeO_2_ catalysts were produced by impregnation using different Cu precursors, such as acetate, chloride, nitrate or sulphate. The samples were calcined at 500 and 800 °C and their physicochemical properties were determined. The results showed that CuO was the dominant Cu phase in all cases. CuO/CeO_2_ prepared from copper acetate and calcined at 500 °C presented the best catalytic results due to the better dispersion of CuO as well as enhanced CuO–CeO_2_ synergistic effects [22]. In addition, Avgouropoulos et al. presented the synthesis of copper-ceria catalysts by a hydrothermal method employing citric acid as a chelating agent, sodium hydroxide as the precipitating agent and different copper precursors (nitrate salt and metallic ring). The catalysts were tested for CO oxidation reaction in the temperature range of 25–400 °C, at atmospheric pressure, using a fixed-bed reactor system. Finally, Avgouropoulos group proved that (i) the catalyst prepared with metallic ring as the copper precursor, named CuCe–CR, achieved CO conversion higher than 99.5% at 215 °C compared to the catalyst prepared with nitrate salt as the copper precursor (CuCe–CN) which showed similar catalytic efficiency at ~245 °C and (ii) high catalytic activity depends on the nature of atomically dispersed Cu^2+^ clusters and not on the specific surface area and concentration of oxygen vacancies [23].

**Figure 1:**
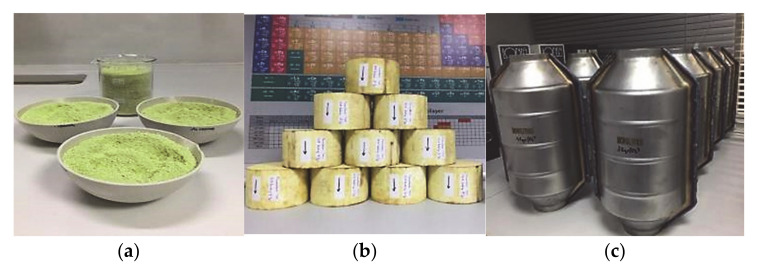
(**a**) Preparation of large-scale PROMETHEUS catalysts (1 kg); (**b**) PROMETHEUS impregnated monoliths; (**c**) PROMETHEUS full-scale ready to install catalysts inside metallic canister.

As a result, due to the reported ability of copper to catalyze oxidation reactions, it has been investigated as an active metal in the synthesis of Three-Way Catalytic converters (TWC). Although many efforts have been made in the field of Automotive industry, PROMETHEUS is the first catalyst produced initially in lab-scale and subsequently in full scale (Figure 1), designed-based on the synergistic effects between copper, noble metals and the high ionic conductivity or oxygen storage capacity of the ceramic carrier, in order to achieve high catalytic activity [24]. It is a disruptive technology since EURO regulations have become more and more severe regarding the use of PGMs (Pt, Pd, Rh).

In 1991 Bedford et al. presented and patented the synthesis of copper-based catalyst supported on Cerium oxide (CeO_2_) and Aluminum oxide particles (Al_2_O_3_) and its use in the catalytic flue gas treatment systems. The efficiency of the supported catalysts was measured under fresh (conversion: 76% HC, 97% CO, 2% NO) and aged conditions (conversion: ~50% HC, ~60% CO, ~2% NO) close to stoichiometric A/F ratio, 14.6 [25]. Later, a copper catalyst supported on CeO_2_, containing small amounts of one noble metal, as enhancer has been presented by Shore et al. Its use was limited to selective oxidation of carbon monoxide in gas streams containing high-concentration of hydrogen [26]. On the other hand, copper presents low activity regarding the reduction of NO in low temperatures. In 1999, Park et al. described the production of a series of catalysts consisting of mixed oxides (oxides of rare earth elements, alkalis, alkaline earth metals with transition metal oxides and noble metals) supported on zeolite catalyst (ZSM-5) [27]. As referred in the patent, the catalysts can be used for the selective catalytic reduction of NO_x_ by using hydrocarbon (olefins or paraffins) as a reducing agent in oxygen excess (lean-burn conditions). More specific, by using copper ion exchanged Cu-ZSN-5 zeolite the conversion of nitrogen monoxide had reached an efficiency point of (91.3%) at 420 °C [27]. Similarly, catalytic efficiency of CuO/CeO_2_ and CuO/γ-Al_2_O_3_ catalysts was measured for the NO + CO reaction. Below 200 °C the loading amount of catalyst, the nature of the support and the dispersion of copper oxide species on the surface of the support have a strong effect on the catalytic activity of catalysts. Only after the temperature was increased to 300 °C, NO was completely converted to N_2_ over the catalysts [28].

PROMETHEUS is a low-cost catalyst for reducing of the three toxic pollutants present in gasoline engine flue gases; CO, C_x_H_y_ and NO_x_, which consists of copper (Cu) and two noble metals, palladium (Pd) and rhodium (Rh). In the present work, the catalytic activity of PROMETHEUS full-scale catalysts towards the oxidation of CO and CH_4_ and the reduction of NO is presented and discussed extensively. The synthesis and characterization of the catalyst have already been studied and published in our previous work [24]. Furthermore, commercial three-way catalysts which represent the performance of catalysts suitable for Euro III (catalyst OEM III), Euro IV (catalyst OEM IV) and Euro V/VI (catalyst OEM V/VI) protocol, have been used as benchmark. Their catalytic performance has been studied for comparison reasons. The two full-scale PROMETHEUS monolithic catalysts with loading 5 g PGMs/ft^3^ and 15 g PGMs/ft^3^ correspond to EURO III catalyst and EURO IV/V/VI, respectively. The catalytic experiments took place on an in-house Synthetic Gas Bench apparatus (SGB) under selected simulated gasoline engine exhaust conditions under both reducing (λ = 0.99) and oxidizing (λ = 1.03) conditions. All the catalysts were tested after hydrothermal ageing treatment at 1050 °C for 4 h under 10% H_2_O in air flow. Finally, both fresh and aged samples of the tested PROMETHEUS catalyst presented excellent catalytic activity and characteristics similar or even improved compared with the commercially available catalysts bearing higher loading on PGMs.

## 2. Materials and Methods

### 2.1. Tested Catalysts

PROMETHEUS catalytic washcoat production which consists of 2 wt% Cu, Pd and Rh nanoparticles supported over Ce_0.68_Zr_0.32_O_2_ inorganic carrier (PROM2) and its deposition on monolithic cordierites achieving the production of full-scale PROMETHEUS catalysts have been presented in our previous work [24]. In more details, for the production of full-scale PROMETHEUS monolithic catalysts, ceramic cordierite-based monoliths (Mg, Fe)_2_Al_4_Si_5_O_18_) were impregnated to produce full scale catalysts ready to be installed to vehicles.

**Figure 2:**
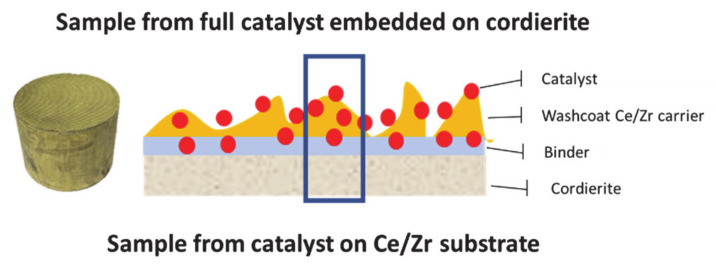
Configurations of the samples used on this study (full scale catalyst).

Initially, the bulk characteristics of ceramic monolith were determined followed by drying at 90 °C. Then, an aqueous slurry containing the appropriate amount of catalytic powder PROM2 and γ-Al_2_O_3_ boehmite (SASOL, GmbHbinder, Hamburg, Germany) was prepared at room temperature under stirring by adjusting the pH value at pH 7 units with 25% aq. NH_4_OH buffer solution (Kalogeropoulos Industrial, Piraeus, Greece). The monolith was impregnated in the produced slurry repeatedly and dried at 90 °C between the impregnation repetitions in different orientation each time, in order to avoid capillary forces phenomena in the cells of the cordierite. The impregnated monolith was finally calcinated at 500 °C for 1 h (heating ramp. 10 °C·min^−1^) and then blown with air, in order for the non-coated particles to be removed. The metal loading of the monolith was determined by the weight of the monolith before and after the impregnation. In case the final weight of coated monolith was not the desired one, the wash coating step was repeated. For this study, catalytic activity experiments took place with samples obtained from the full-scale catalyst, including catalytic nanoparticles, washcoat and the substrate of the cordierite (Figure 2). This approach was followed in order for the catalytic measurement results to be comparable to the benchmark original catalysts.

**Figure 3:**
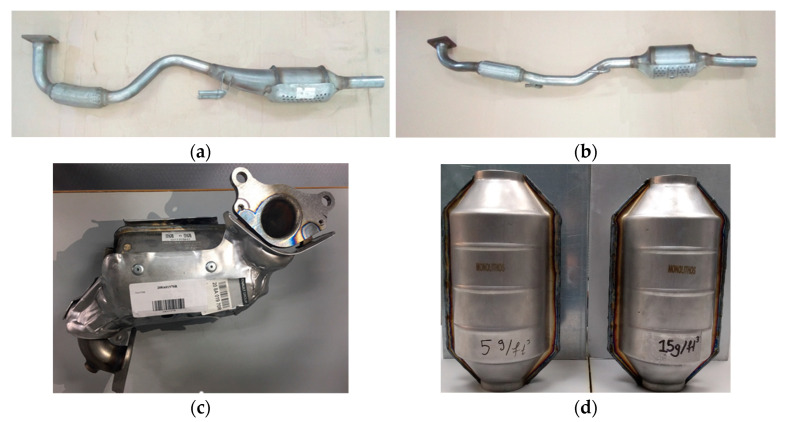
(**a**) OEM EURO III exhaust spare part for VW Polo 1.4 Lt; (**b**) OEM EURO IV exhaust spare part for VW Polo 1.4 Lt; (**c**) OEM EURO V/VI exhaust spare part for Renault Meganne 1.2 Lt Turbo; (**d**) PROMETHEUS Full Scale Monolithic Catalysts (5–15 g/ft^3^).

Herein, the two produced full-scale PROMETHEUS catalysts with 5 g PGMs/ft^3^ and 15 g PGMs/ft^3^ loading, named 5PROM2 and 15PROM2, respectively and the commercial catalysts OEM III (VW Group Polo), OEM IV (VW Group Polo) and OEM V/VI (Renault Meganne) used as benchmark (Figure 3), were tested and evaluated towards the oxidation of CO and CH_4_ and reduction of NO in the synthetic gas bench apparatus. The commercial TWCs were selected as benchmark catalysts and represent the performance of a catalyst suitable for Euro III (catalyst OEM III), Euro IV (catalyst OEM IV) and Euro V/VI (catalyst OEM V/VI) protocol. In addition, PROMETHEUS catalysts incorporate 85% less PGMs compared to the above original catalysts; 5PROM2 for EURO III applications (compared with 32 g/ft^3^ PGMs of the original catalyst) and 15PROM2 for EURO IV/V/VI applications (compared to more than 100 g/ft^3^ PGMs of the original catalyst). All the full-scale catalysts (PROMETHEUS full scale catalysts and commercial catalysts) were characterized by X-ray Fluorescence Spectroscopy in order the existence and subsequently the concentration of PGMs (Pt, Pd, Rh) and copper to be determined prior to the catalytic experiments and by Optical Microscopy for the structure analysis.

### 2.2. X-ray Fluorescence Spectroscopy

Prior to the catalytic experiments X-ray fluorescence spectroscopy (XRF, Vanta Olympus, Waltham, MA, USA) was applied in catalysts samples (PROMETHEUS full scale catalysts and commercial catalysts) for qualitative and quantitative analysis (Table 1). As seen from Table 1, commercial catalysts OEM III, OEM IV and OEM V/VI consist of PGMs (Pd and Rh), while full-scale PROMETHEUS catalysts consist of PGMs (Pd and Rh) and Cu. In more detail, the full-scale catalysts were grinded to pieces and then subjected to milling and sieved in order for particle size <125 μm powder to be obtained (Planetary Ball Mill Machine LITH-XQM-0.4, Lith Machine Ltd., Xiamen, Fujian, China). Finally, the obtained powder was dried at 120 °C for 2 h. The metal content of the catalysts was determined by the analysis of powder received by the corresponding catalysts. For the analysis, the samples were prepared in the form of pressed powder inside polyethylene cups where the appropriate amount of catalyst powder was found to be approximately 5 g for each sample. The analysis of the samples was carried out by measuring each sample with 10 repeated scans, of 90 s each. In order for higher accuracy to be obtained in XRF measurement, the XRF instrument (Vanta Olympus, Waltham, MA, USA) was calibrated for each metal separately, additionally to the internal calibration of the instrument. In the case of precious metals (Pt, Pd and Rh), thirteen automotive catalyst samples with varied metal concentration were used for calibration. On the other hand, in case of copper, commercial samples with known metal concentrations were used for calibration (OREAS Pty Ltd. CRMs, Bayswater, North Vic, Australia). The nominal concentration value was already measured by ICP-MS method. Pt has been calibrated in the loading range of 600–2800 ppm, Pd in the loading range of 1270–2730 ppm, Rh in the loading range of 230–330 ppm and Cu in the loading range of 0.07–12% [29].

### 2.3. Optical Microscopy

**Figure 4:**
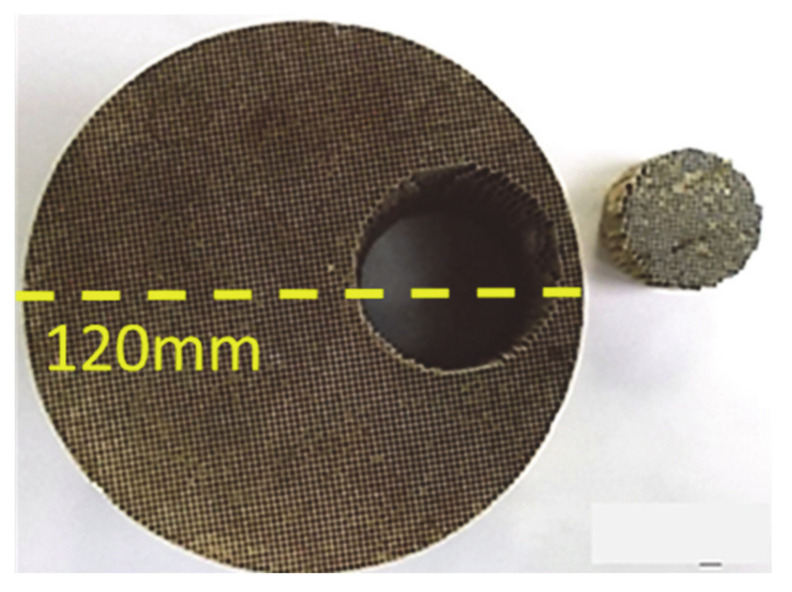
Carrot extraction from 15PROM2 catalyst.

**Figure 5:**
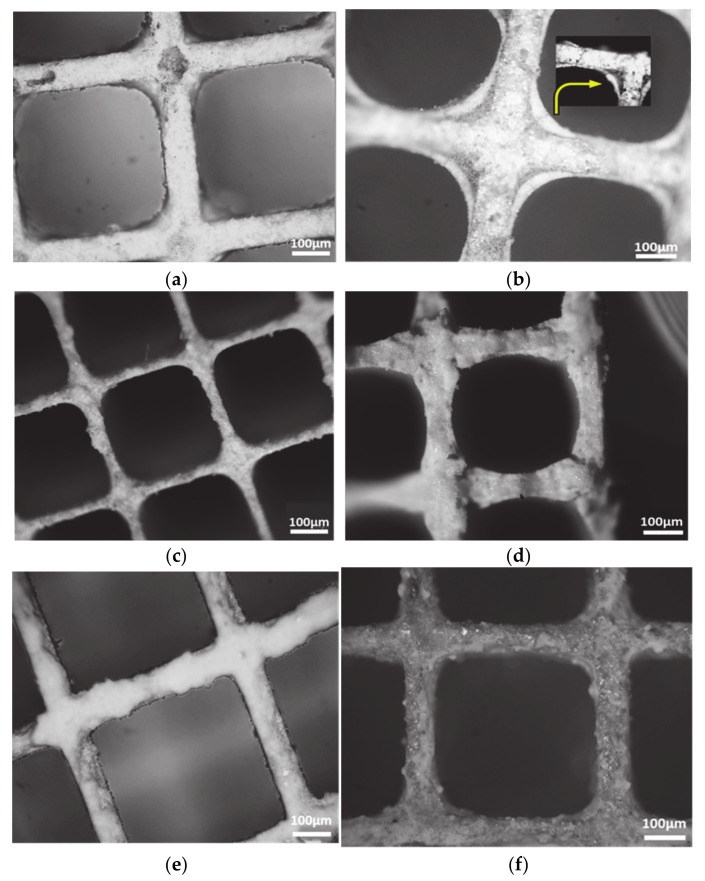
Optical microscopy cross-section images (5×) of the catalysts: (**a**) OEM III; (**b**) OEM V/VI (inlet image 10×); (**c**) Primary catalyst of OEM V/VI; (**d**) Second catalyst of OEM V/VI; (**e**) 5PROM2; (**f**) 15PROM2.

The sample for the structure analysis of the catalysts was prepared according to the following procedure, a piece of each catalyst in shape of carrot (dimensions: d: 4.4 cm, l: 7.6 cm) was extracted from the main cordierite (Figure 4) and then each sample has been cross-sectionally cut so that the monolith cells can be observed. Optical microscopy, using a metallurgical microscope by AmScope (ME520 series, Irvine, CA, USA) equipped with a microscope digital camera 14 MP ultrafine color was used. Each sample was placed under the objective lenses in order to be moved in the vertical direction to focus and obtain a resolution suitable for measuring the thickness of the wash-coat. In Figure 5, the optical microscopy cross-section images of the benchmark catalysts OEM III, OEM IV, OEM V/VI and the PROMETHEUS Full Scale monolithic catalysts 5PROM2 and 15PROM2 at 5 × optical zoom are presented.

As shown, in case of OEM III, only one coating layer can be distinguished on the walls of the cordierite matrix; corresponding to the catalyst distributed on the wash coat, having thickness of approximately 50–120 µm (Figure 5a), while for OEM IV catalyst two coating layers can be distinguished on the walls of the cordierite matrix; a thin internal layer covering mostly the “dead volume” at the corner zones and a second thin catalyst layer on top of the first one. Both coatings seem to be uniform, with thickness of approximately 70–100 µm (Figure 5b). In the examined sample of the primary catalyst OEM V/VI the thickness of the catalytic surface layer was approximately 20–70 µm, while the interlayer is approximately 10–20 µm (Figure 5c). On the other hand, in the sample of the second catalyst OEM V/VI the thickness of the catalytic surface layer is approximately 20–100 µm, while the interlayer is approximately 10–50 µm (Figure 5d). Finally, in the piece of 5PROM2 (5 g/ft^3^) and 15PROM2 (15 g/ft^3^) catalysts the thickness of the catalytic surface layer is 20–50 μm (Figure 5e) and 50–100 μm (Figure 5f), respectively, which proves that are similar to the corresponding commercial catalysts.

### 2.4. Synthetic Gas Bench (SGB)

**Figure 6:**
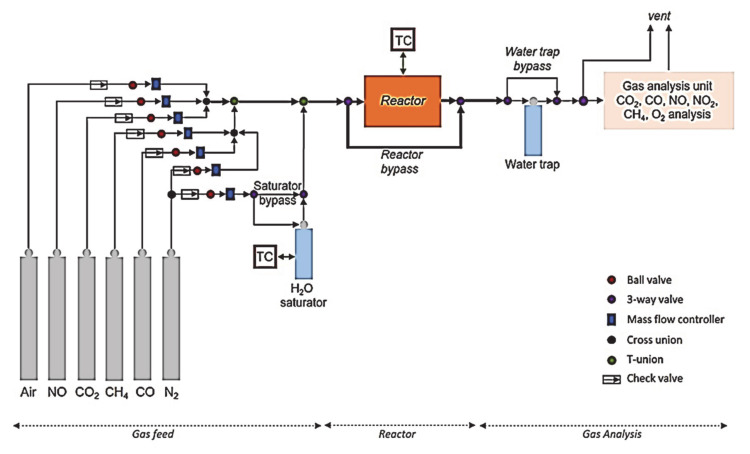
Schematic representation of the experimental setup used for the catalytic activity test of catalysts.

**Figure 7:**
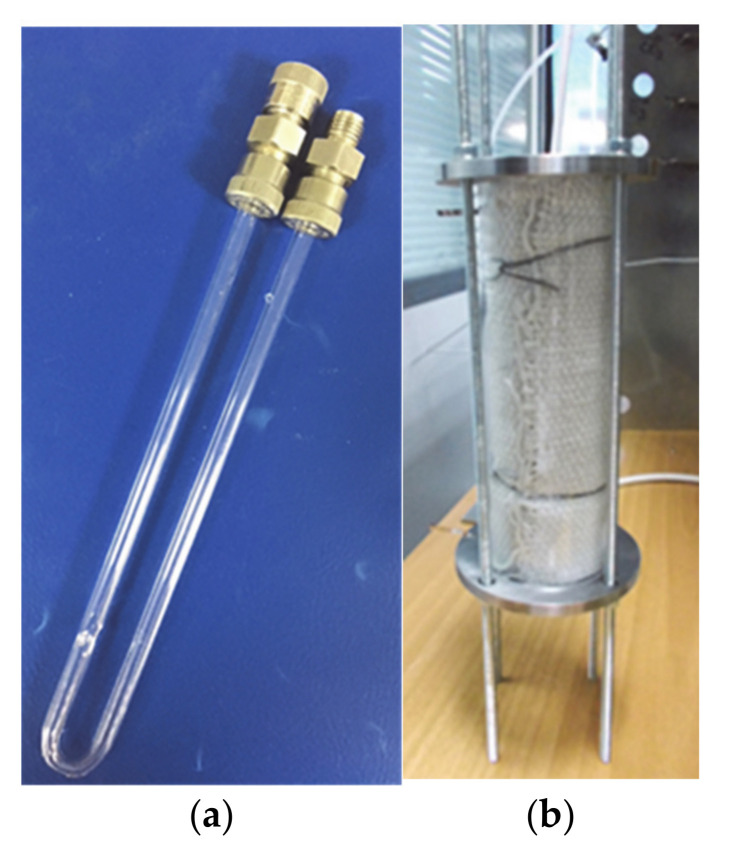
(**a**) U-shaped quartz reactor for activity testing of catalyst powders; (**b**) tube experimental electrical furnace.

The catalytic activity of both produced and commercial catalysts was tested in an in-house synthetic gas bench (SGB) apparatus. The lab-scale SGB device was designed and built in MONOLITHOS (Athens, Greece) premises. A schematic representation of the experimental setup is shown in Figure 6, where three regions can be distinguished: the gas feed, the reactor and the gas analysis. The gas feed region contains the reactant gases (certified standards) of Air, NO, CO_2_, CH_4_, CO (each gas was diluted in N_2_) and pure N_2_ for balance, the mass flow controllers for mixing the gases and the thermostated water saturator for steam introduction to the gas mixture. The reactor region contains a thermostated furnace and a U-shaped quartz reactor (Figure 7), while the gas analysis region contains the water trapping system and the gas analysis system.

**Figure 8:**
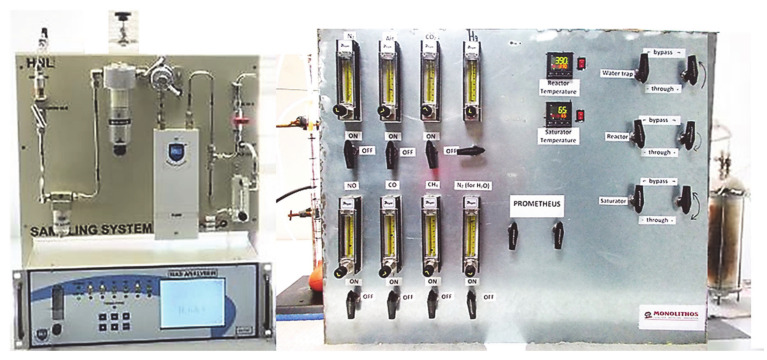
Catalyst activity lab-scale testing setup (SGB apparatus).

Moreover, in Figure 8, a picture of the experimental setup is presented, where the reactor furnace stands on the right of the control panel. The gas analyzer (GA-200 PVT, HNL Ltd., Mumbai, India) enables the simultaneous analysis of CO_2_, CO, O_2_, NO, CH_4_ and NO_2_ and is used downstream the reactor region. Need to be mentioned that the formation of N_2_O was found to be in low selectivity <50 ppm in the range temperature of 250–300 °C and as a result this product has not been discussed in the present study. The analysis of gases is carried out through an automated sampling system for a fixed sampling time.

The experimental setup enables fast on-site performance screening of catalyst powders under selected simulated gasoline engine exhaust conditions. It should be noted that CH_4_ was used for the simulation of mixture in a real exhaust gas of a petrol engine as it is the major constituent of total hydrocarbon emissions (THC) [30]. As a result, catalytic activity tests were performed both under reducing (λ = 0.99) and oxidizing conditions (λ = 1.03) (lambda sensor λ = A/F; A: air, F: Fuel), in the temperature range between 100–525 °C. In both conditions, the total flow rate was 300 sccm, which corresponded to a gas hourly space velocity of 50.000 h^−1^. The composition of the two examined gas mixtures applied in the catalytic activity tests are summarized in Table 2.

### 2.5. Catalyst Ageing Procedure

Catalyst ageing was performed in all presented herein catalysts according to certified protocol. According to the protocol, each sample was heated up to 1050 °C for 4 h under 10% H_2_O air flow. A high temperature calcination furnace was used equipped with an atmospheric air electric pump for air introduction to the heating chamber. Upstream the furnace, the air stream was passing through a thermostated water saturator operating at 46 °C, for the introduction of 10% H_2_O in air mixture into the furnace chamber. Finally, catalytic activity tests were performed both under reducing and oxidizing conditions (λ = 0.99 and λ = 1.03, respectively), in the temperature range between 100 and 550 °C. In both conditions the total flow rate was 300 sccm, which corresponded to a gas hourly space velocity of 50,000 h^−1^. The composition of the two examined gas mixtures applied in the catalytic activity tests are summarized in Table 2.

## 3. Results

### 3.1. Catalytic Activity of Fresh Catalysts

Initially, the catalytic efficiency of fresh commercial-benchmark and produced PROMETHEUS catalysts were tested in the synthetic gas bench (SGB) apparatus, simulating the operation of an internal combustion engine. The experimental conditions were selected based on certified testing protocol and were kept constant in all experiments. Catalytic activity tests were performed under both reducing and oxidizing conditions (λ = 0.99 and λ = 1.03, respectively), in the temperature range between 100 and 550 °C. The composition of the two examined gas mixtures applied in each condition are summarized in Table 2. The fresh samples of catalysts are expected to show the best catalytic performance, as they represent a brand-new catalyst of zero mileage.

#### 3.1.1. Catalytic Efficiency Comparison of OEM III with 5PROM2 Full Scale Catalyst (84.4% Substitution of PGMs with Copper NanoParticles)

**Figure 9:**
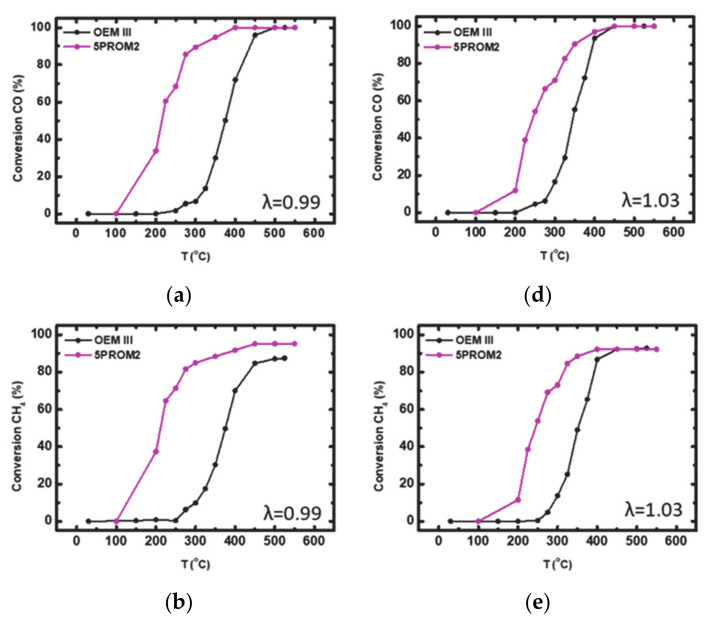

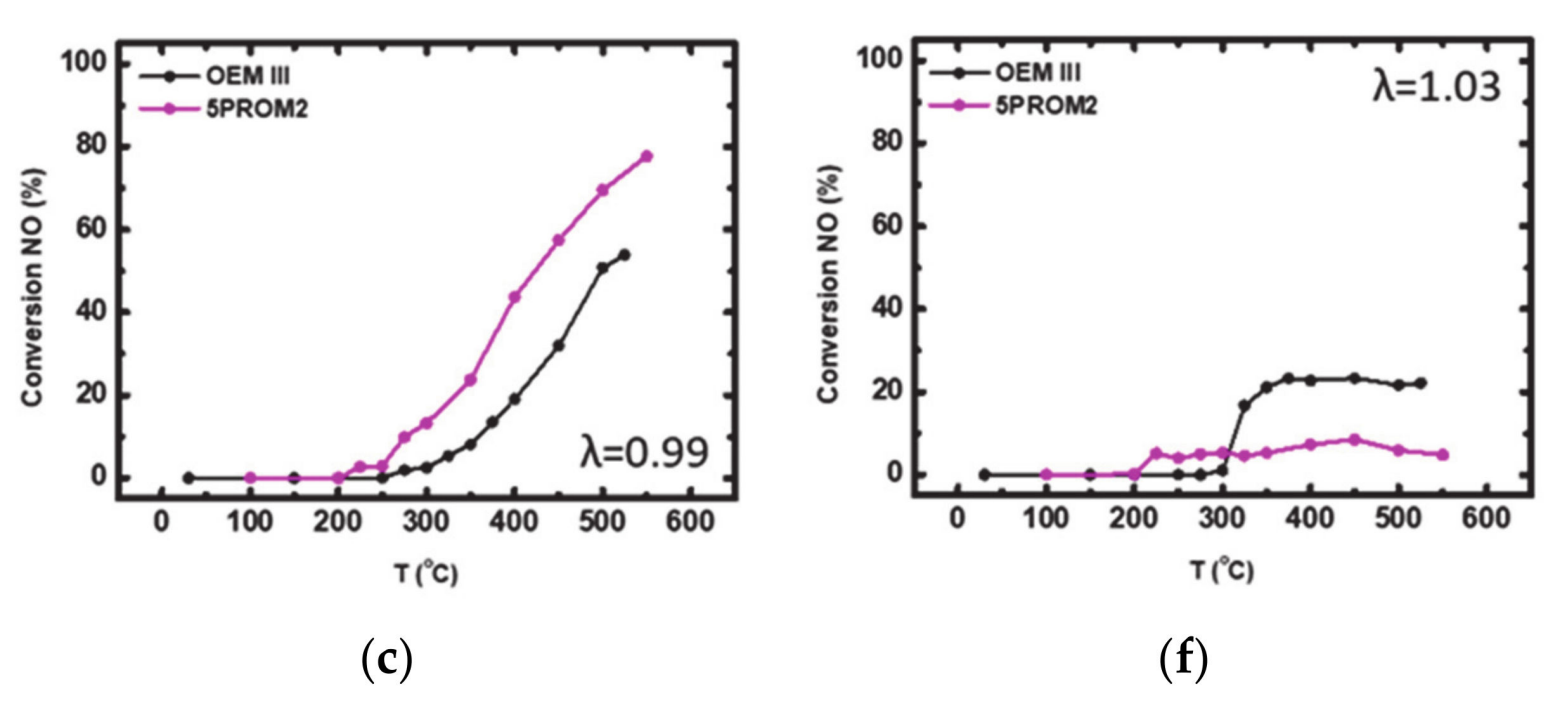
Light-off curves for the (%) conversion of: (**a**) CO; (**b**) CH_4_; (**c**) NO under rich-burn conditions (λ = 0.99); (**d**) CO; (**e**) CH_4_; (**f**) NO under lean-burn conditions (λ = 1.03) over commercial catalyst OEM III and PROMETHEUS Full Scale monolithic catalyst 5PROM2.

In Figure 9 the light-off curves (conversion vs. temperature) for CO and CH_4_ oxidation and for NO reduction in both rich- and lean-burn catalytic conditions (λ = 0.99 and λ = 1.03) over the fresh commercial catalyst OEM III and PROMETHEUS full scale monolithic catalyst 5PROM2 are presented. Important activity indication values for catalyst characterization for rich- and lean-burn catalytic conditions are summarized in Table 3 and Table 4, respectively.

As mentioned above, the catalysts OEM III and 5PROM2 are suitable for Euro III emission standard. According to the results in rich-burn conditions (λ = 0.99) the two catalysts were active at T > 260 °C and T > 200 °C, respectively. It is proved that CO oxidation efficiency reached ~100% in each case (Figure 9a), while CH_4_ oxidation efficiency was limited to 87% for OEM III and 95% for 5PROM2 catalyst (Figure 9b). Furthermore, NO reduction reached 54% at ~525 °C for OEM III catalyst and 78% at T ~550 °C for 5PROM2 catalyst (Figure 9c). On the other hand, under lean-burn conditions (λ = 1.03) catalysts were active at T > 270 °C and T > 200 °C, respectively. The CO oxidation efficiency reached ~100% over the two catalysts (Figure 9d), while CH_4_ oxidation reached 93% for OEM III and 92% for PROMETHEUS catalyst 5PROM2 (Figure 9e). Finally, NO reduction was low (OEM III: 23%, 5PROM2: 8%) possibly due to surface oxidation of Rh nanoparticles of the catalysts (Figure 9f). As a result, comparing the catalytic efficiency of the two catalysts, PROMETHEUS full scale monolithic catalyst 5PROM2, presents similar and in some cases higher catalytic activity in lower light-off temperatures (T50 and T90) compared to the commercial catalyst OEM III by significantly substitution of PGMs (Pd and Rh) with copper nanoparticles. Especially, in case of CO and CH_4_ conversion (%), 5PROM2 catalyst presents much lower values of T50 and T90 in both rich-burn and Lean-burn conditions with similar yield value (%) to OEM III catalyst.

#### 3.1.2. Catalytic Efficiency Comparison of OEM IV and OEM V/VI with 15PROM2 Full Scale Catalyst (85.1% Substitution of PGMs with Copper NanoParticles)

**Figure 10:**
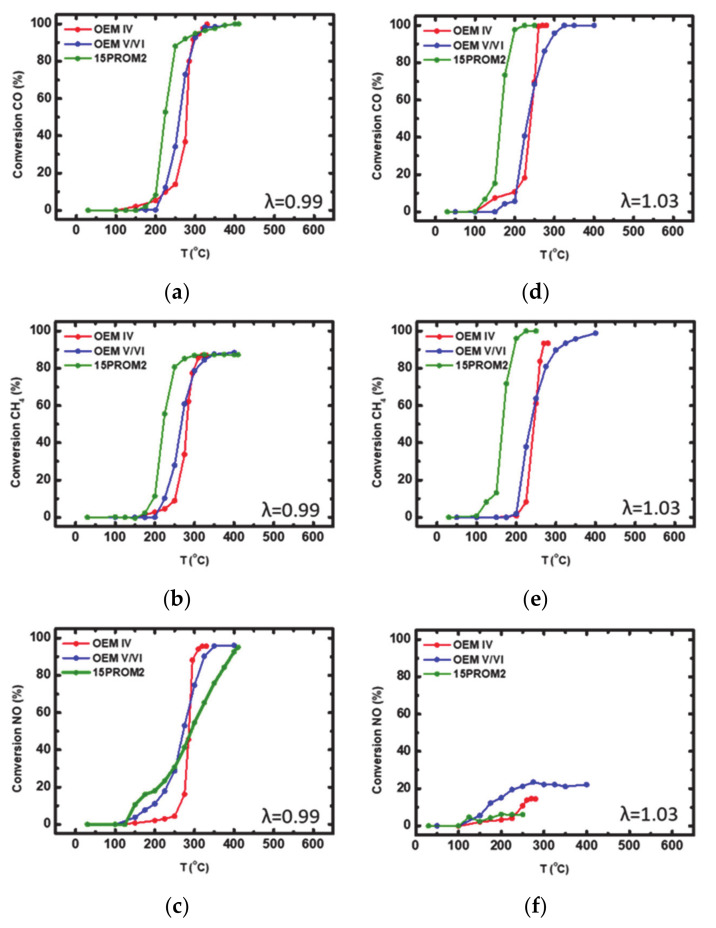
Light-off curves for the (%) conversion of: (**a**) CO; (**b**) CH_4_; (**c**) NO under rich-burn conditions (λ = 0.99); (**d**) CO; (**e**) CH_4_; (**f**) NO under lean-burn conditions (λ = 1.03) over commercial catalysts OEM IV, OEM V/VI and PROMETHEUS Full-scale monolithic catalyst 15PROM2.

The two commercial catalysts OEM IV, OEM V/VI and the produced full-scale PROMETHEUS monolithic catalyst, named 15PROM2 were also tested under both rich-burn (λ = 0.99) and lean-burn (λ = 1.03) conditions for the abatement of CO, CH_4_ and NO. The corresponding light-off curves are shown in Figure 10 and catalytic activity values are presented in Table 5 and Table 6, respectively.

Under rich-burn conditions OEM IV, OEM V/VI and 15PROM2 catalysts were active at T > 250, T > 200 and T > 190 °C, respectively. CO oxidation efficiency reached 100% in each case, while CH_4_ oxidation efficiency was reached 87, 88 and 87%, respectively (Figure 10a,b). It is worth noting that the reduction efficiency of NO was recorded at 96% (T ~320 °C) over OEM IV catalyst, at 96% (T > 350 °C) over OEM V/VI catalyst and 96% (T ~410 °C) over 15PROM2 catalyst (Figure 10c). On the other hand, under lean-burn conditions (λ = 1.03), 100% conversion of CO was occurred over the two commercial catalysts (OEM IV and OEM V/VI), while CH_4_ oxidation efficiency reached 93% and 99%, respectively. PROMETHEUS full scale catalyst 15PROM2 presented full CO and CH_4_ conversion (Figure 10d,e). Finally, in all cases poor NO reduction reaction efficiency was observed (OEM IV: 15%, OEM V/VI: 22% and 15PROM2: 6%, Figure 10f). It should be stressed out that PROMETHEUS full-scale catalyst (15PROM2) presents lower light-off temperatures comparative to the corresponding commercial catalysts, with high conversion (%) values of the three toxic gases, CO, CH_4_, NO even though it consists of lower concentration of PGMs (Pd and Rh). This proves the successful replacement of PGMs (85.1%) by Cu.

#### 3.1.3. Comparison of the Tested Fresh Catalysts

The impact of the strictest emission limits on the emissions of vehicles tailpipe pollutants is reflected on the development of the catalytic converters, suitable for the corresponding Euro protocol. A higher PGM content is observed on catalysts of the latest Euro-IV (108 g/ft^3^) and Euro-V/VI (101 g/ft^3^) protocols (catalysts OEM-IV and OEM-V/VI) compared with catalysts of Euro-III protocol (32 g/ft^3^, catalyst OEM-III). This increased PGM content has a significant impact on the catalytic activity and properties of the catalysts, as indicated by the experimental results (Table 3, Table 4, Table 5 and Table 6). Catalysts that meet the requirements of strictest Euro protocols (Euro IV and Euro V/VI, catalysts OEM IV and OEM V/VI, respectively) with higher metal content are characterized by low light-off temperatures which are achieved for all oxidation and reduction reactions. This improved catalytic activity is observed for both rich-burn and lean-burn conditions.

PROMETHEUS catalysts exhibited high and comparable catalytic activity to the corresponding commercial TWC catalysts, proving that copper can successfully replace a part of PGMs. The most interesting results are observed for the oxidation of CH_4_ over 15PROM2 catalyst, which is achieved at lower temperatures for both rich-burn (T_50_ ≈ 220) and lean-burn (T_50_ ≈ 160 and T_90_ ≈ 190) conditions. This performance is better even than a commercially available EURO-V/VI catalyst (T_50_ ≈ 237 and T_90_ ≈ 305 for lean-burn conditions) and indicates the beneficial effect of the copper presence. Only on the reduction of NO the values of T_50_ ≈ 290 and T_90_ ≈ 390 are higher compared to the corresponding achieved values of EURO-IV (T_50_ ≈ 286 and T_90_ ≈ 300) and EURO-V/VI (T_50_ ≈ 270 and T_90_ ≈ 325) catalysts.

As Rh is the metal that catalyze the NO reduction, the higher temperature values of PROMETHEUS catalyst 15PROM2 are linked with the low Rh content. Further improvement to the NO reduction activity of PROMETHEUS, can take place with the change of the ratio of oxidizing/reduction metals of the catalyst. While for the commercial catalysts, the Pd/Rh ratio varies from 82/18 to 92/8, for PROMETHEUS the specific ratio (Cu + Pd over Rh is 96.5/3.5). A change in the specific ratio (reducing Pd in favor of Rh) keeping the same overall PGM concentration could enhance the already very good performance of PROMETHEUS catalyst over the commercial ones.

### 3.2. The Effect of Ageing in Catalytic Activity

Automotive three-Way Catalysts, the most widely employed gasoline exhaust after treatment devices, are able to perform a simultaneous reduction of more than 90% of the engine-out CO, HC and NO_x_ emissions and are usually operated in a wide temperature range from 150 to 1000 °C in oscillating conditions around stoichiometric air-to-fuel ratio, fluttering between oxidant and reducing environment and in presence of water vapor in a stream. Operation of TWC under extremely harsh conditions (T > 850 °C) can cause deactivation of the catalyst, a phenomenon called thermal ageing, presenting a decreased conversion efficiency and higher light-off temperatures. Thermal ageing is linked to the modification of the washcoat structure, called sintering, following by a loss of active surface area via structural modification of the porous support with a decrease of surface area of the carrier [31,32]. As a result, stringent emissions standards require high efficiency in emissions abatement and catalyst performance at high mileage [33].

Based on the above, OEMs and aftermarket TWC manufactures use different ageing methods such as, vehicle ageing, engine bench ageing or laboratory ageing process, with the latter to be the most widespread over the last years since it is proved to be a time-saving method by presenting high efficiency. Vehicle ageing method is actually close to real road driving condition but, on the other hand, it is time-consuming, it takes more manpower and the cost is high. Furthermore, according to recent publications of Centre Research Fiat and University of Torino, after applying comparative tests of laboratory ageing, real on vehicle aging and engine bench aging it was proved the similar results of gases conversion (%) with no significant variations [34,35,36].

**Figure 11:**
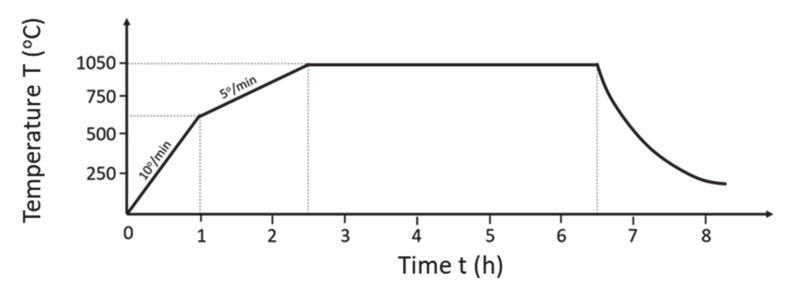
Ageing process heating protocol. Atmosphere: 10% H_2_O in air.

In this work a hydrothermal laboratory ageing protocol was used for the produced full-scale PROMETHEUS monolithic catalysts (5PROM2 and 15PROM2) and the commercial catalysts (OEM III, OEM IV, OEM V/VI) which is able to overcome the limitations of accelerated engine bench tests ensuring the simulation of a catalytic converter mileage under real life conditions. According to the protocol each catalyst was heated up to 1050 °C for 4 h under 10% H_2_O in air flow and then their performance was evaluated under rich-burn (λ = 0.99) and lean-burn (λ = 1.03) conditions for the abatement of CO, CH_4_ and NO. (Figure 11). Need to be mentioned that, the researchers of the Centre Research FIAT (CRF) and University of Torino concluded that hydrothermal ageing at 1100 °C for 7 h simulate Light Temperature for CO and HC of 80,000 real kilometers.

The authors have, also, applied a catalyst hydrothermal ageing protocol according to international legislation standards (the legislation indicates ageing simulating 160,000 Km of real run) at 850 °C for 16 h to the catalysts of this study indicating mild deactivation (for this reason the results are not presented, since they do not offer further conclusions for the performance of the catalysts as compared with the fresh ones). Under these circumstances the ageing protocol near to melting point of copper (1085 °C) was used in order to have a more severe environment for the copper-based PROMETHEUS catalysts. A partial Cu leaching was observed during the ageing process without the catalytic efficiency of the catalysts being affected severely.

#### 3.2.1. Catalytic Efficiency of AOEM III with A5PROM2 Full Scale Catalyst (84.4% Substitution of PGMs with Copper NanoParticles)

**Figure 12:**
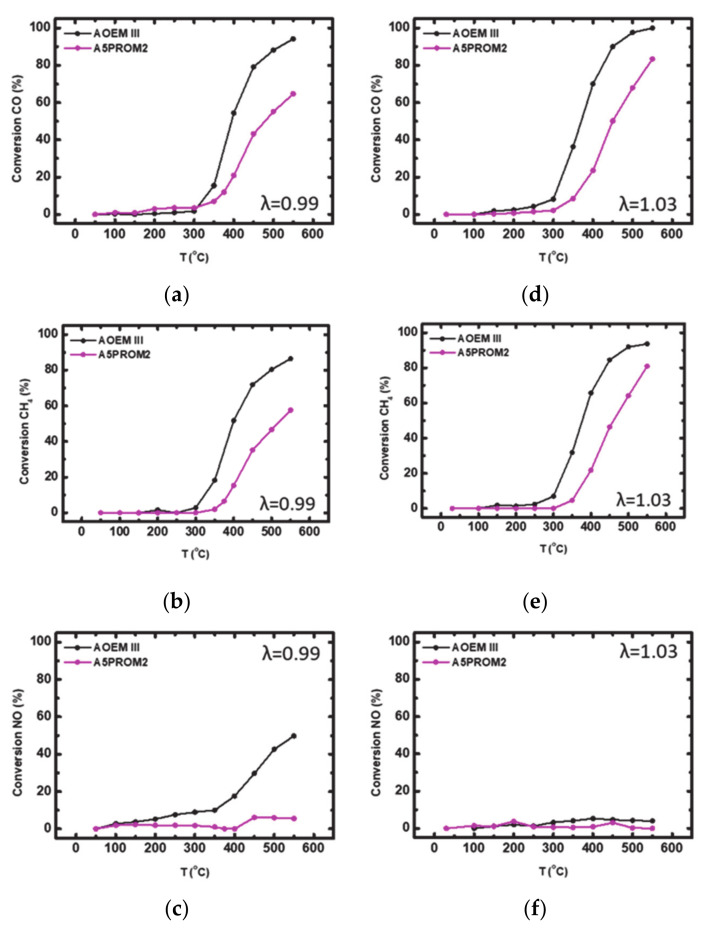
Light-off curves for the (%) conversion of: (**a**) CO; (**b**) CH_4_; (**c**) NO under rich-burn conditions (λ = 0.99); (**d**) CO; (**e**) CH_4_; (**f**) NO under lean-burn-conditions (λ = 1.03) over aged commercial catalyst AOEM III and PROMETHEUS Full Scale monolithic catalyst 5PROM2.

The aged at 1050 °C for 4 h (according to the above-mentioned protocol) commercial catalyst AOEM III and PROMETHEUS full scale monolithic catalyst 5PROM2, were tested under both rich-burn (λ = 0.99) and lean-burn (λ = 1.03) conditions, for the abatement of the three toxic gases CO, CH_4_ and NO. Corresponding light-off curves are shown in Figure 12. Important activity indication values for catalysts characterization are summarized in Table 7 and Table 8, respectively.

As shown in Table 7 and Table 8, a significant degradation in catalytic activity was observed after catalyst ageing procedure. According to the results, in rich-burn conditions (λ = 0.99) the two catalysts were active at T > 300 °C and T > 370 °C, respectively. It is proved that CO oxidation efficiency reached 94 and 65% in each case (Figure 12a), while CH_4_ oxidation efficiency was limited to 87 and 58%, respectively (Figure 12b). Furthermore, NO reduction reached 50% at 550 °C for AOEM III catalyst and only 6% for A5PROM2 catalyst (Figure 12c). On the other hand, under lean-burn conditions (λ = 1.03) catalysts were active at T > 300 °C and T > 350 °C, respectively. CO conversion reached 100% over AOEM III and 83% over A5PROM2 catalyst (Figure 12d), while CH_4_ oxidation reached 94% for AOEM III and 81% for A5PROM2 catalyst (Figure 12e). Finally, NO reduction was very low (AOEM III: 5%, A5PROM2: 0), possibly due to surface oxidation of Rh nanoparticles of the catalysts (Figure 12f).

#### 3.2.2. Catalytic Efficiency of AOEM IV and AOEM V/VI with A15PROMW Full Scale Catalyst (85.1% substitution of PGMs with Copper NanoParticles)

**Figure 13:**
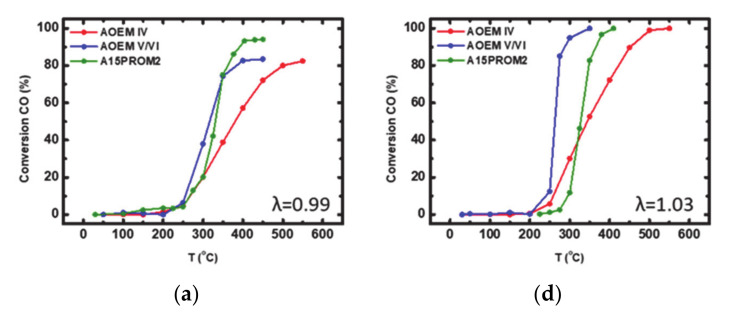

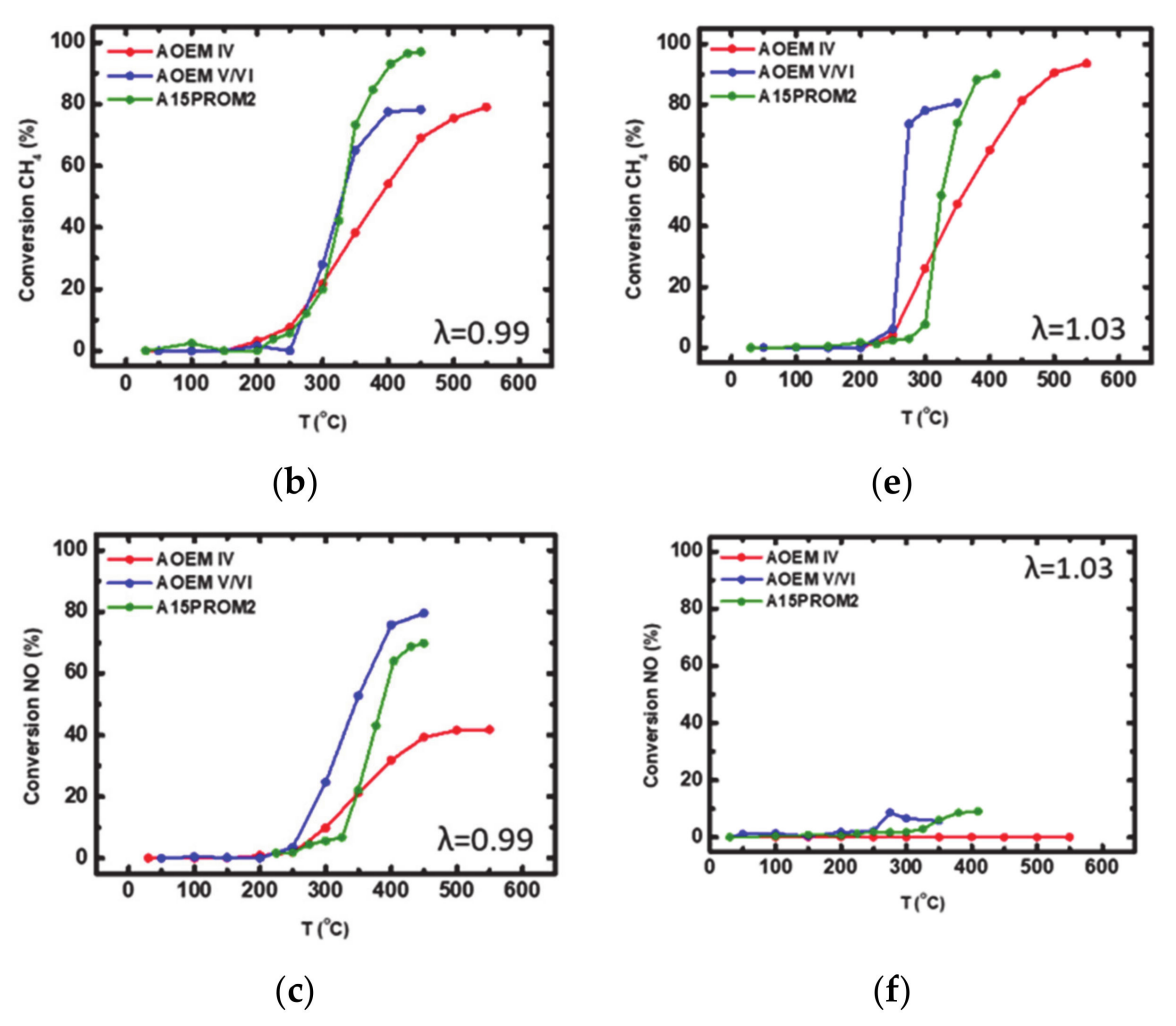
Light-off curves for the conversion (%) of: (**a**) CO; (**b**) CH_4_; (**c**) NO under rich-burn conditions (λ = 0.99); (**d**) CO; (**e**) CH_4_; (**f**) NO under lean-burn-conditions (λ = 1.03) over aged commercial catalysts AOEM IV, OEM V/VI and PROMETHEUS Full Scale monolithic catalyst 15PROM2.

The aged commercial catalysts AOEM IV, AOEM V/VI and PROMETHEUS full-scale catalyst 15PROM2 were tested under both rich-burn (λ = 0.99) and lean-burn (λ = 1.03) conditions, for the abatement of CO, CH_4_ and NO. Corresponding light-off curves are shown in Figure 13. Important activity indication values for catalysts characterization are summarized in Table 9 and Table 10, respectively.

After ageing at 1050 °C, catalytic activity was detected only above 250, 250 and 270 °C under rich-burn conditions over AOEM IV, AOEM V/VI and A15PROM2 catalysts, respectively. CO oxidation efficiency reached 82, 83 and 94% (Figure 13a), while CH_4_ oxidation efficiency limited to 79, 78 and 97%, respectively (Figure 13b). NO reduction reached 42% at 550 °C for AOEM IV, 80% at 450 °C for AOEM V/VI and 70% at ~400 °C for A15PROM2 catalyst (Figure 13c). It can be noticed that A15PROM2 catalyst presents similar light-off temperature and maximum efficiency behavior compared with the original EURO V/VI benchmark. Furthermore, under lean-burn conditions (λ = 1.03) the catalysts were active above 250, 230 and 300 °C, respectively. CO conversion (%) reached to 100% in each case (Figure 13d), while CH_4_ conversion (%) was found equal to 94, 81 and 90%, respectively (Figure 13e). A15PROM2 presented similar maximum efficiency behavior with Original EURO V/VI benchmark, while a higher by 70 °C light-off temperature was observed for the former, probably due to the severe ageing protocol that was implemented. The melting point of copper is 1085 °C (very near to the ageing temperature of 1050 °C) implying that part of the metal could be passing in liquid form, lowering the copper content of the catalyst and thus, the synergistic effect between copper and PGMs is decreased.

## 4. Discussion

The progress of the catalytic converters technology resulted in new type of catalysts, able to meet the requirements of the strictest Euro protocols for automotive emissions. These strictest emission limits are related with the protection of the environment, the prevention of climate change and the improvement of air quality, especially in the large urban centers, through the decrease of emission of the transport sector. Apart from the environmental point, the economic point of view is also important. The last generation catalytic converters require increased content of Platinum Group Metals (PGMs) in order to meet strict emission criteria and directions. This fact subsequently leads to elevated cost of the final product and dependence on the countries that produced the raw material. The research presented herein suggests an alternative approach that combines an effective catalytic system and lower cost. This performance is achieved by the use of copper, which according to the presented technology can successfully replace a significant amount of PGMs in the catalytic converters. According to the experimental results, the presented PROMETHEUS catalysts can successfully meet the Euro III and Euro V/VI emission protocols. More specific, catalyst 5PROM2 containing 5 gr/ft^3^ of PGMs presents similar or better catalytic activity when it is compared with OEM III containing 32 gr/ft^3^ PGMs. Furthermore, catalyst 15PROM2 containing 15 gr/ft^3^ of PGMs presents similar or better catalytic activity when it is compared with OEM IV and OEM V/VI containing more than 100 gr/ft^3^ PGMs. Overall, 85% reduction of PGM loading has been achieved by incorporating PROMETHEUS Substitution Technology with similar or better catalytic efficiency results. The reduction of NO under lean-burn conditions is the only exception, which can be attributed to the lower ratio of oxidation metals (Cu and Pd) and reduction metal (Rh) 28/1 as compared to the original catalysts (Pd/Rh ratio between 10.5/1 to 4.5/1).

The pronounced higher activity of PROMETHEUS catalyst that contains ~85% less PGM (or ~7 times less PGM), can be possibly attributed to synergistic phenomena between Cu and PGM metal nanoparticles and to high oxygen storage capacity of the selected Ceria-Zirconia ceramic support at low temperatures. The latter results in decoration of the metal particles surface with back spillover oxygen ionic species acting as in-situ activity promoters. According to the above and, based on the literature, we may propose the following mechanism. The high activity and stability of the catalyst is mainly attributed to the existence of Cu (I) species in the system and the destabilization of Cu (II) species, both due to e- transfer effects from the Rh, Pd metals to Cu and strong metal-support in ter-action phenomena. Oxygen ion species vacancies on the wash-coat support are favored, which results in weakening of the interaction between the adsorbed electronegative species from the gas phase to the surface metal oxide species. In addition, the ability of Ce (III) to get oxidized by steam in the temperature range 300–500 °C and produce H_2_(g) which can then cause the reduction of Cu(I) to Cu, plays an important role. The latter step is of particular importance since the metal Cu formed therein serves the reduction of Ce (IV) thus completing the Ce (III)/Ce (IV) oxidation cycle. In the above-described synergy, it is also worth mentioning the recorded steam chemi-sorption on the surface of CuPt bimetallic catalysts, thus contributing to the critical step of Ce (III) oxidation and the formation of H_2_ [37,38,39]. In our future work, extensive mechanistic study will be take place in order to ensure the above mentioned and proposed mechanism and to fully explain the high catalytic efficiency of PROMETHEUS catalyst.

## 5. Conclusions

The two full-scale PROMETHEUS catalysts with loading 5 g PGMs/ft^3^ and 15 g PGMs/ft^3^ corresponding to EURO III catalyst and EURO IV/V/VI were tested for the abatement of toxic gases CO, CH_4_ and NO. Furthermore, three commercial catalysts suitable for Euro III (catalyst OEM III), Euro IV (catalyst OEM IV) and Euro V/VI (catalyst OEM V/VI) protocol, have been used as benchmark and their catalytic efficiency has been tested for comparison reasons under the same catalytic conditions. The produced PROMETHEUS catalysts and the commercial catalysts were then aged at 1050 °C for 4 h and were evaluated as catalysts for the oxidation of CO and CH_4_ and the reduction of NO.

According to the results, 15PROM2 and A15PROM2 catalysts present in most cases better results compared to 5PROM2 and A5PROM2 catalysts due to higher PGMs loading, 15 g PGMs/ft^3^. On the other hand, both fresh and aged PROMETHEUS catalysts present similar and, in some cases, better catalytic activity compared to corresponding commercial catalysts although they consist of 85% less PGMs, thus opening a new path for the production of catalytic converters.

## 6. Patents

European Patent of Prometheus: copper and noble metal polymetallic catalysts for engine flue gas treatment. EP3569309. Applicant Monolithos Catalysts and Recycling Ltd., Inventor: Iakovos Yakoumis.

## Figures and Tables

**Table 1:** PGMs and copper loading for commercial Three-Way Catalysts TWCs (benchmark catalysts) and PROMETHEUS full scale catalysts produced.

Abbreviation	Company	Model	Motor	Euro	Cu (ppm/%wt.)	Pd (ppm/%wt.)	Rh (ppm/%wt.)	PGM Loading (g/ft^3^) ^1^
5PROM2	MONOLITHOS	Universal	Up to 1.6 Lt	III	820 0.082	280 0.028	40 0.004	5
OEM III	VW Group	Polo	1.4 Lt	III	—	1392 0.1392	295 0.0295	32
OEM IV	VW Group	Polo	1.4 Lt	IV	—	4872 0.4872	306 0.0306	108
OEM V/VI	Renault	Meganne	1.2 Lt turbo	V/VI	—	5642 0.5642	471 0.0471	101
15PROM2	MONOLITHOS	Universal	Up to 1.6 Lt	VI	2320 0.232	780 0.078	110 0.011	15

^1^ mass and geometric volume of the monolithic were calculated before crushing.

**Table 2:** Selected gas mixtures concentrations for λ = 0.99 and λ = 1.03.

λ Factor Values	Gas Component					
	CO (%)	CO_2_ (%)	O_2_ (%)	NO (ppm)	CH_4_^1^ (ppm)	H_2_O (%)
Rich-burn conditions λ = 0.99	1	12	0.91	800	2500	10
Lean-burn conditions λ = 1.03	1	12	0.95	800	2500	10

^1^ CH_4_ was used for the simulation of mixture in a real exhaust gas of a petrol engine.

**Table 3:** Comparison of tested catalysts OEM III and 5PROM2 under rich-burn conditions (λ = 0.99).

Catalyst	CO Oxidation				CH_4_ Oxidation				NO Reduction			
	T_50_ (°C)	T_90_ (°C)	T_99_ (°C)	Max. Efficiency (%)	T_50_ (°C)	T_90_ (°C)	T_99_ (°C)	Max. Efficiency (%)	T_50_ (°C)	T_90_ (°C)	T_99_ (°C)	Max. Efficiency (%)
OEM III	375	437	490	100%	375	—	—	87%	500	—	—	54%
5PROM2	215	306	388	100%	212	375	—	95%	425	—	—	78%

**Table 4:** Comparison of tested catalysts OEM III and 5PROM2 under lean-burn conditions (λ = 1.03).

Catalyst	CO Oxidation				CH_4_ Oxidation				NO Reduction			
	T_50_ (°C)	T_90_ (°C)	T_99_ (°C)	Max. Efficiency (%)	T_50_ (°C)	T_90_ (°C)	T_99_ (°C)	Max. Efficiency (%)	T_50_ (°C)	T_90_ (°C)	T_99_ (°C)	Max. Efficiency (%)
OEM III	345	390	440	100%	350	420	—	93%	—	—	—	23%
5PROM2	240	350	430	100%	240	375	—	92%	—	—	—	8%

**Table 5:** Comparison of tested catalysts OEM IV, OEM V/VI and 15PROM2 under rich-burn conditions (λ = 0.99).

Catalyst	CO Oxidation				CH_4_ Oxidation				NO Reduction			
	T_50_ (°C)	T_90_ (°C)	T_99_ (°C)	Max. Efficiency (%)	T_50_ (°C)	T_90_ (°C)	T_99_ (°C)	Max. Efficiency (%)	T_50_ (°C)	T_90_ (°C)	T_99_ (°C)	Max. Efficiency (%)
OEM IV	280	293	330	100%	280	—	—	87%	286	300	—	96%
OEM V/VI	260	296	370	100%	265	—	—	88%	270	325	—	96%
15PROM2	220	260	370	100%	220	—	—	87%	290	390	—	96%

**Table 6:** Comparison of tested catalysts OEM IV, OEM V/VI and 15PROM2 under lean-burn conditions (λ = 1.03).

Catalyst	CO Oxidation				CH_4_ Oxidation				NO Reduction			
	T_50_ (°C)	T_90_ (°C)	T_99_ (°C)	Max. Efficiency (%)	T_50_ (°C)	T_90_ (°C)	T_99_ (°C)	Max. Efficiency (%)	T_50_ (°C)	T_90_ (°C)	T_99_ (°C)	Max. Efficiency (%)
OEM IV	240	258	260	100%	244	268	—	93%	—	—	—	15%
OEM V/VI	233	285	320	100%	237	305	400	99%	—	—	—	22%
15PROM2	160	190	210	100%	160	190	215	100%	—	—	—	6%

**Table 7:** Comparison of aged tested catalysts OEM III and 5PROM2 under rich-burn conditions (λ = 0.99).

Catalyst	CO Oxidation				CH_4_ Oxidation				NO Reduction			
	T_50_ (°C)	T_90_ (°C)	T_99_ (°C)	Max. Efficiency (%)	T_50_ (°C)	T_90_ (°C)	T_99_ (°C)	Max. Efficiency (%)	T_50_ (°C)	T_90_ (°C)	T_99_ (°C)	Max. Efficiency (%)
AOEM III	400	515	—	94	400	—	—	87	550	—	—	50
A5PROM2	482	—	—	65	514	—	—	58	—	—	—	6

**Table 8:** Comparison of aged tested catalysts OEM III and 5PROM2 for rich-burn conditions (λ = 1.03).

Catalyst	CO Oxidation				CH_4_ Oxidation				NO Reduction			
	T_50_ (°C)	T_90_ (°C)	T_99_ (°C)	Max. Efficiency (%)	T_50_ (°C)	T_90_ (°C)	T_99_ (°C)	Max. Efficiency (%)	T_50_ (°C)	T_90_ (°C)	T_99_ (°C)	Max. Efficiency (%)
AOEM III	370	450	525	100	370	490	—	94	—	—	—	5
A5PROM2	449	—	—	83	460	—	—	81	—	—	—	0

**Table 9:** Comparison of aged tested catalysts AOEM IV, AOEM V/VI and 15PROM2 under rich-burn conditions (λ = 0.99).

Catalyst	CO Oxidation				CH_4_ Oxidation				NO Reduction			
	T_50_ (°C)	T_90_ (°C)	T_99_ (°C)	Max. Efficiency (%)	T_50_ (°C)	T_90_ (°C)	T_99_ (°C)	Max. Efficiency (%)	T_50_ (°C)	T_90_ (°C)	T_99_ (°C)	Max. Efficiency (%)
AOEM IV	390	—	—	82	390	—	—	79	—	—	—	42
AOEM V/VI	315	—	—	83	325	—	—	78	340	—	—	80
A15PROM2	330	390	—	94	330	395	—	97	390	—	—	70

**Table 10:** Comparison of aged tested catalysts AOEM IV, AOEM V/VI and 15PROM2 under lean-burn conditions (λ = 1.03).

Catalyst	CO Oxidation				CH_4_ Oxidation				NO Reduction			
	T_50_ (°C)	T_90_ (°C)	T_99_ (°C)	Max. Efficiency (%)	T_50_ (°C)	T_90_ (°C)	T_99_ (°C)	Max. Efficiency (%)	T_50_ (°C)	T_90_ (°C)	T_99_ (°C)	Max. Efficiency (%)
AOEM IV	345	450	500	100	358	495	-	94	—	—	—	0
AOEM V/VI	260	289	345	100	260	—	-	81	—	—	—	9
A15PROM2	330	366	404	100	325	410	-	90	—	—	—	9

## Data Availability

The data presented in this study are available on request from the corresponding author after obtaining permission of authorized person. The data are not publicly available due to data confidentiality resulting from the requirements of the accreditation.
